# Supplementary material for: The inhibitory effects of toothpaste and mouthwash ingredients on the interaction between the SARS-CoV-2 spike protein and ACE2, and the protease activity of TMPRSS2 in vitro
Source: PLoS One. 2021 Sep 17;16(9):e0257705. doi: 10.1371/journal.pone.0257705 (PMC8448299; doi:10.1371/journal.pone.0257705)
Supplement: S1 File — (DOCX) [file pone.0257705.s001.docx]

# S1 File. Supplemental Methods

**The analysis of the effect of ingredient on fluorescent substance using the assay of TMPRSS2 serine protease activity**

Test ingredients and 7-Amino-4-methyl coumarin (2.5 μM final concentration) diluted with assay buffer added into the black 384 well plates. Immediately after dilution, fluorescence intensity was read using the SpectraMax M5 plate reader at excitation of 380 nm and emission of 460 nm. The inhibitory rate of ingredients was calculated as follows: Inhibition (%) = (FI of control – FI of treatment) / FI of control × 100. This assay was performed in triplicate.

## *In vitro* assay of the interaction between the receptor-binding domain of spike protein and ACE2 in assay solutions containing saliva

The interaction between the receptor-binding domain (RBD) of the SARS-CoV-2 spike protein and ACE2 was estimated using Spike S1 (SARS-CoV-2): ACE2 Inhibitor Screening Colorimetric Assay Kit. Saliva was used Saliva - COVID-19 Negative Samples (Unconfirmed) (Lee Biosolutions, Inc, Maryland Heights, MO, USA). Test ingredients and saliva (16.7% (v/v) in this incubate solution) were added to a 96 well plate coated with Spike S1 and incubated for 1 hour. Then, ACE2-Biotin solution was added so that the saliva reached a final concentration (10% (v/v)), and incubated for 1 hour. The following processes were performed according to the materials and methods described in the manuscript. Control data for assay solutions containing saliva and no ingredients were used.

## *In vitro* assay of TMPRSS2 serine protease activity in assay solutions containing saliva

Recombinant human TMPRSS2 (4 μg/mL final concentration) diluted with an assay buffer (50 mM Tris-HCl pH 8.0, 154 mM NaCl), test ingredients and saliva (10% (v/v) final concentration) were added to the 384 well black plate. Then, Boc-Gln-Ala-Arg-MCA (10 μM final concentration) diluted with an assay buffer containing dimethyl sulfoxide (DMSO; 0.1% (w/w) final concentration) was added to induce an enzyme reaction. The following processes were performed according the materials and methods described in the manuscript. Control data for assay solutions containing saliva and no ingredients were used.
