# Supplementary figures and images for: The inhibitory effects of toothpaste and mouthwash ingredients on the interaction between the SARS-CoV-2 spike protein and ACE2, and the protease activity of TMPRSS2 in vitro
Source: PLoS One. 2021 Sep 17;16(9):e0257705. doi: 10.1371/journal.pone.0257705 (PMC8448299; doi:10.1371/journal.pone.0257705)

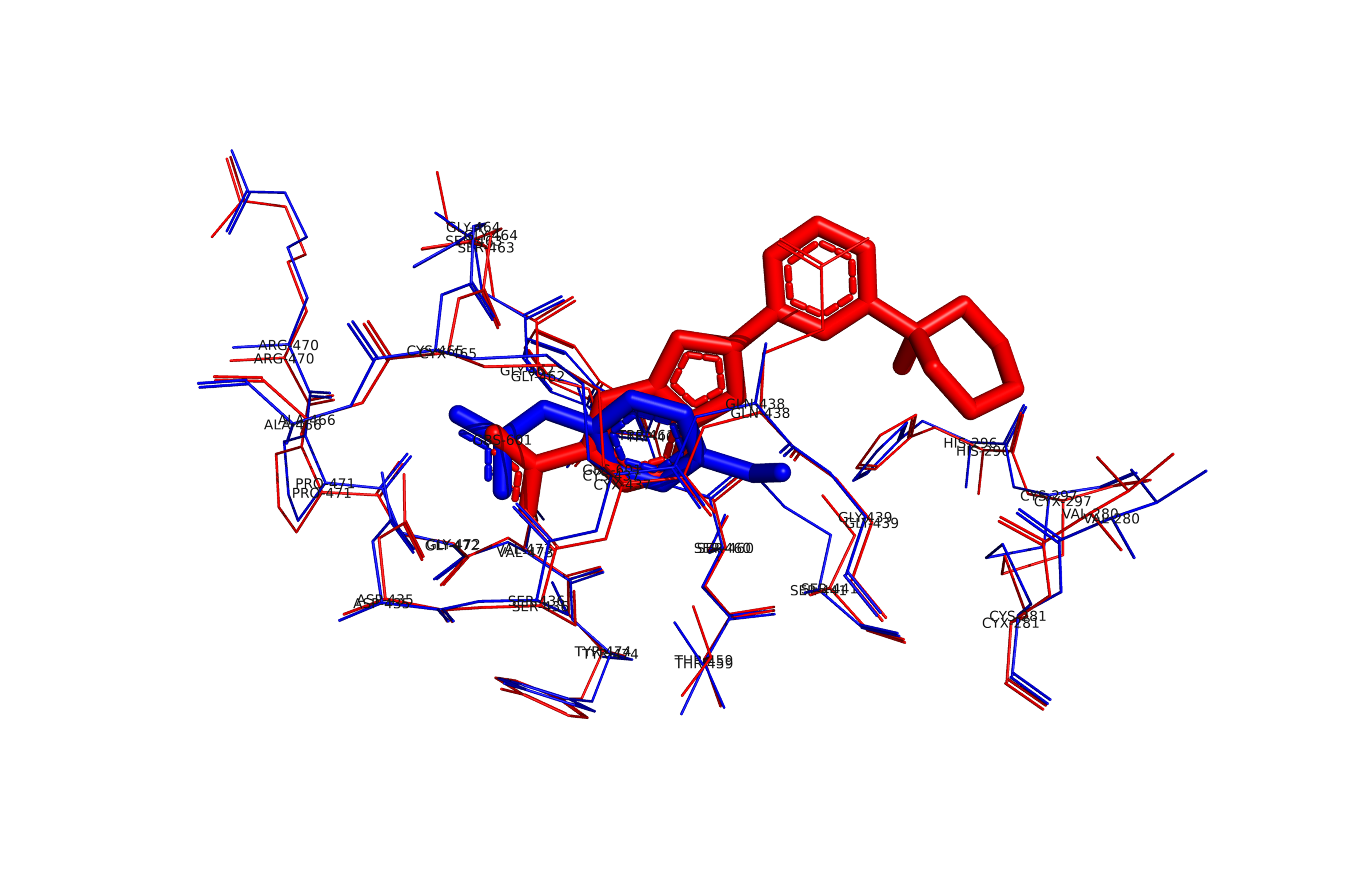

Supplement: S1 Fig — Inhibitor-binding site (the residues located at 5Å from the inhibitor of 5CE1 (shown in red tubes)) was shown in this figure. The Gln438 side-chain atoms of crystal structure (blue) were not observed in 7MEQ. (TIF) [file pone.0257705.s002.tif]

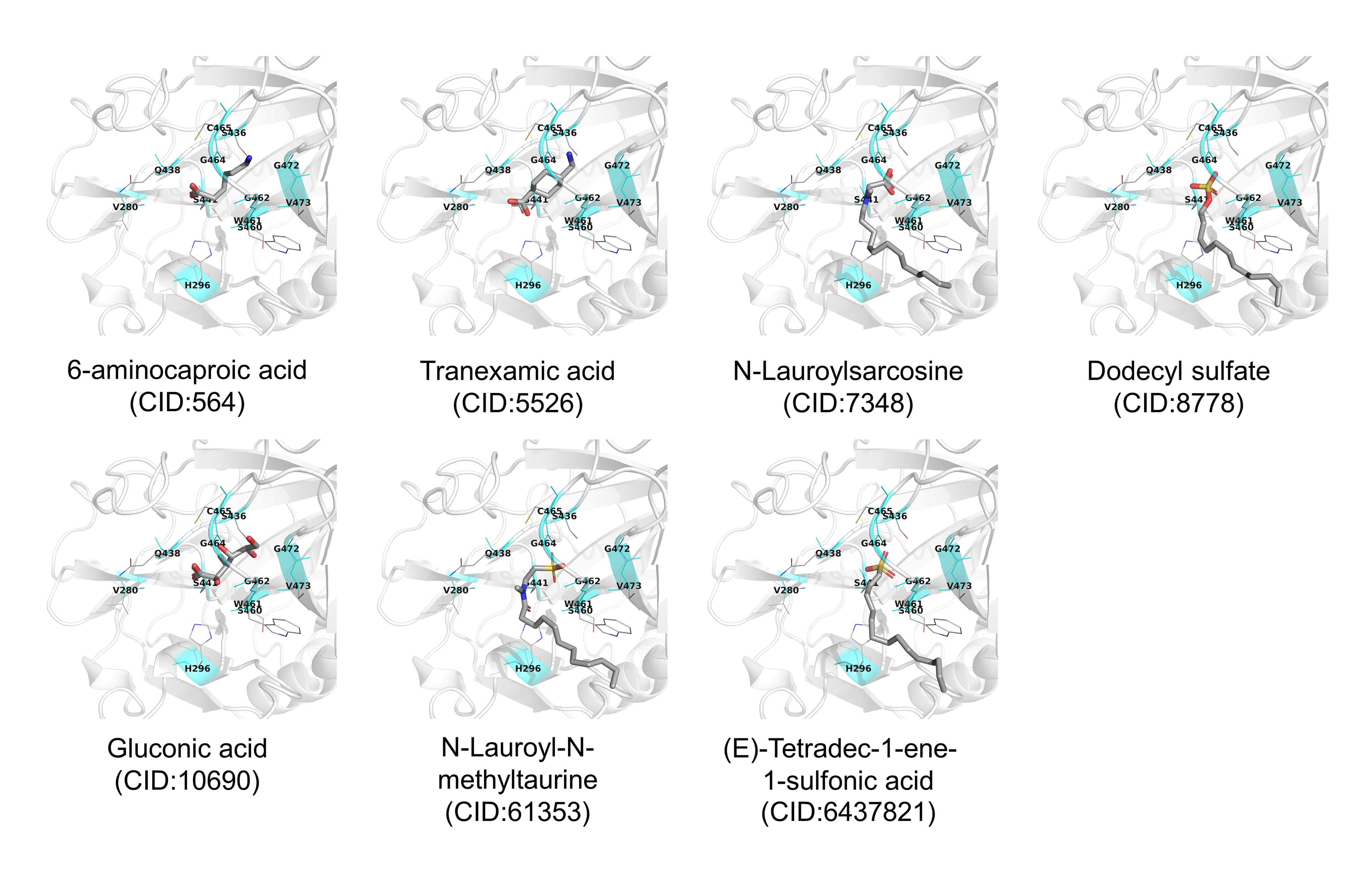

Supplement: S2 Fig — The stable docking mode of test compounds is shown as CPK-colored tubes. The inhibitor-binding site located at 3 Å from all heavy atoms of the crystal inhibitor is shown in cyan color. Amino acid residues located at 3 Å from the inhibitor are shown using thin tubes with labels. Hydrogen atoms are neglected. (TIF) [file pone.0257705.s003.tif]

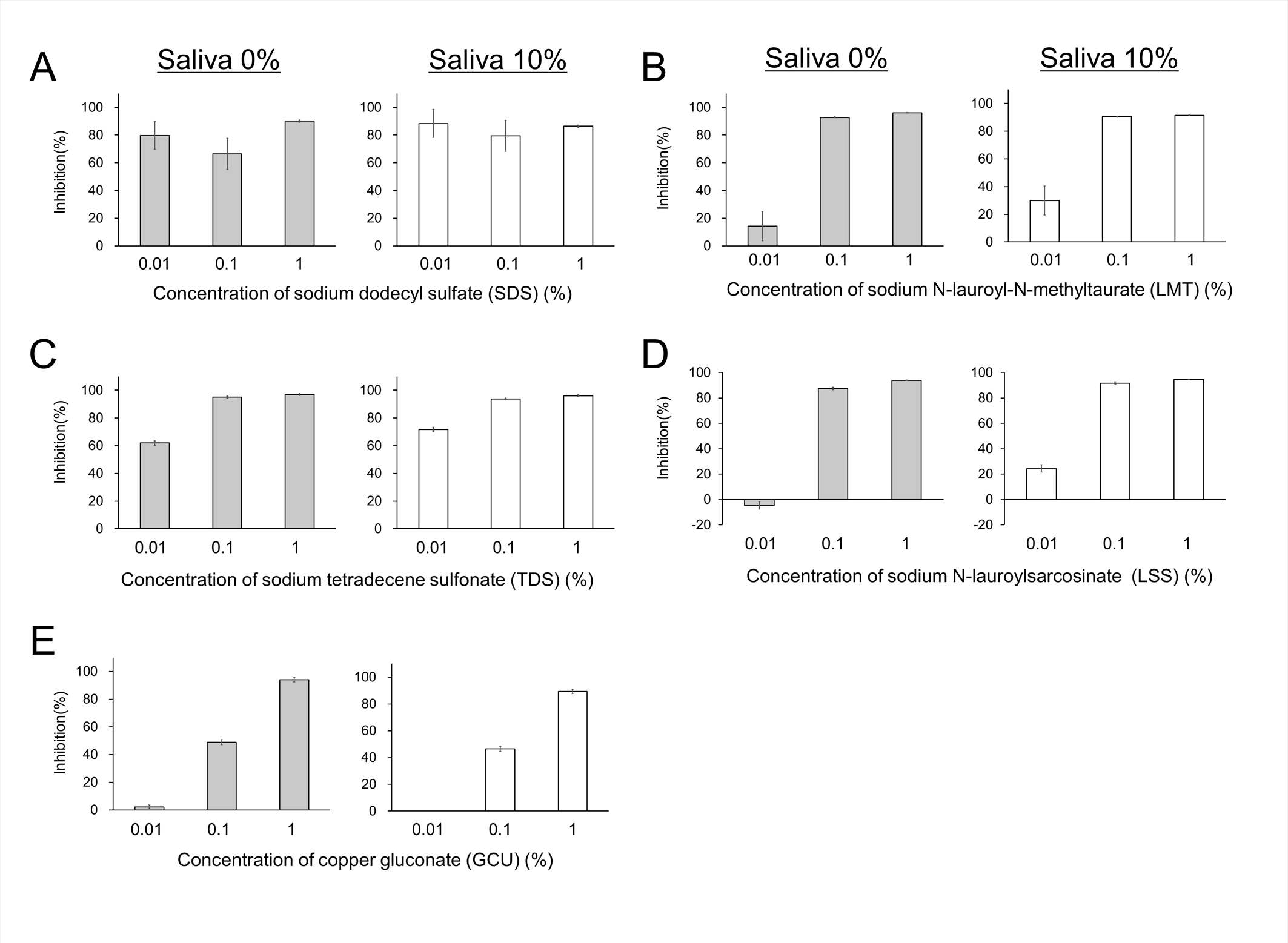

Supplement: S3 Fig — The inhibitory effects in assay solutions with saliva (10% (v/v)) or without saliva (0%(v/v)) of SARS-CoV-2 spike protein-ACE2 interaction by (A) Sodium dodecyl sulfate, (B) Sodium N-lauroyl-N-methyltaurate, (C) Sodium tetradecene sulfonate, (D) Sodium N-lauroylsarcosinate, and (E) Copper gluconate are shown. For the data in the assay solution without saliva, some of the data in Fig 2 are republished to compare the effect with the data in the assay solution with saliva. All data points were expressed as the mean ± SD (n = 3). (TIF) [file pone.0257705.s004.tif]

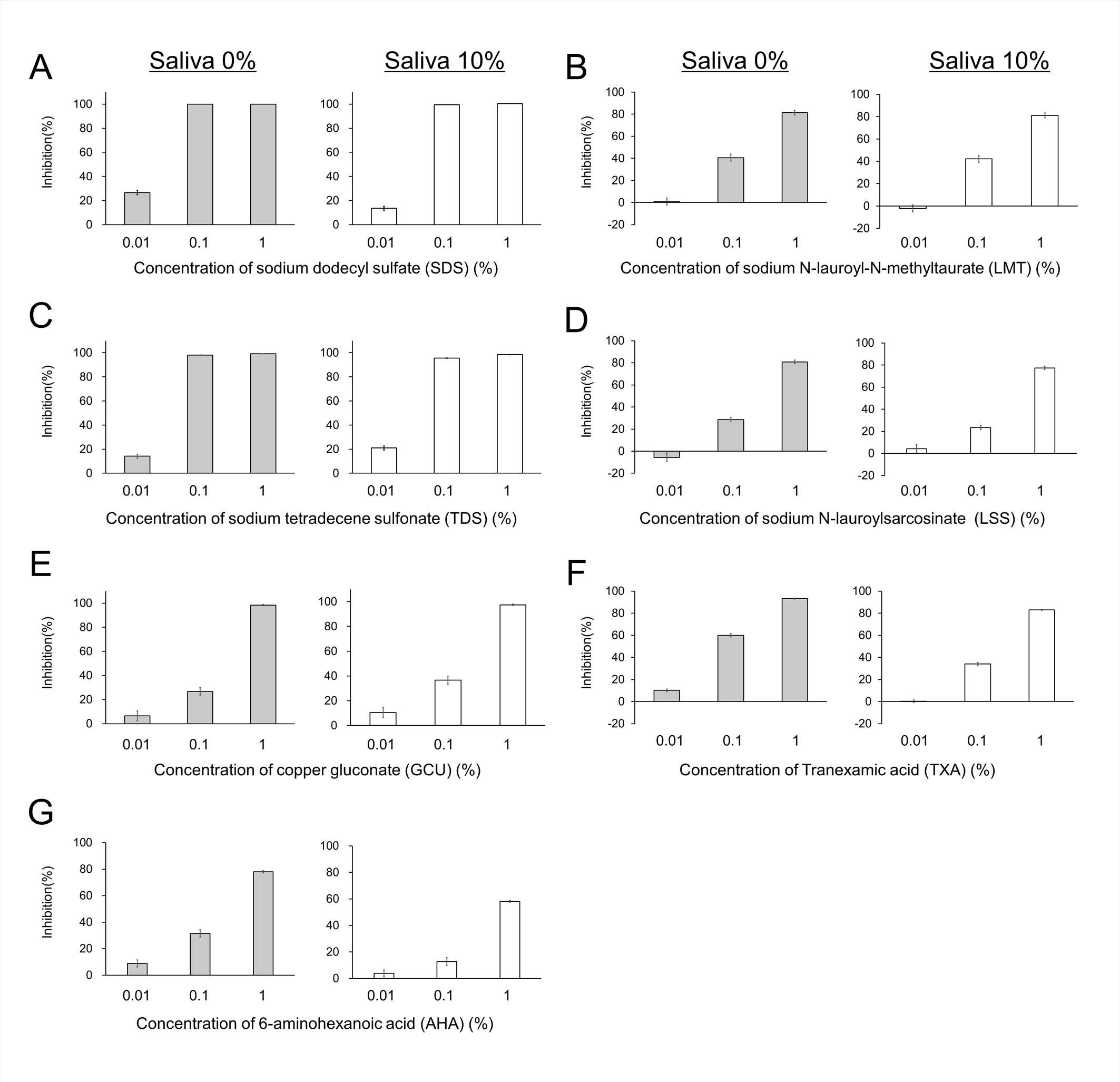

Supplement: S4 Fig — The inhibitory effects in assay solutions with saliva (10% (v/v)) or without saliva (0%(v/v)) of TMPRSS2 serine protease activity by ((A) Sodium dodecyl sulfate, (B) Sodium N-lauroyl-N-methyltaurate, (C) Sodium tetradecene sulfonate, (D) Sodium N-lauroylsarcosinate, and (E) Copper gluconate, (F) Tranexamic acid, and (G) 6-aminohexanoic acid are shown. For the data in the assay solution without saliva, some of the data in Fig 4 are republished to compare the effect with the data in the assay solution with saliva. All data points were expressed as the mean ± SD (n = 3). (TIF) [file pone.0257705.s005.tif]
