# Supplementary material for: The inhibitory effects of toothpaste and mouthwash ingredients on the interaction between the SARS-CoV-2 spike protein and ACE2, and the protease activity of TMPRSS2 in vitro
Source: PLoS One. 2021 Sep 17;16(9):e0257705. doi: 10.1371/journal.pone.0257705 (PMC8448299; doi:10.1371/journal.pone.0257705)
Supplement: S1 Table — (DOCX) [file pone.0257705.s006.docx]

**S1 Table. Effect of ingredient on fluorescent substance (7-Amino-4-methylcoumarin)**

| Ingredients | Abbreviation | Inhibition (%) | |
| --- | --- | --- | --- |
|  |  | average | SD |
| Cetylpyridinium chloride | CPC | 84.53 | 2.80 |
| Sodium saccharin | SAC | 69.82 | 6.40 |
| Copper gluconate | GCU | 24.31 | 2.16 |
| Sodium DL-pyrrolidonecarboxylate  (50% solution) | PCA | 13.81 | 6.50 |
| Tranexamic acid | TXA | 12.57 | 14.63 |
| Sodium cocoamphoacetate (40% solution) | SCP | 9.01 | 5.98 |
| Sodium citrate | CIS | 5.86 | 2.75 |
| Benzyldimethyltetradecylammonium chloride | BZC | 3.64 | 10.77 |
| Calcium 2-glycerophosphate | GPC | 0.92 | 19.19 |
| Polyoxyethylene (15) cetyl ether | PEC | -3.27 | 4.07 |
| Sodium dodecyl sulfate | SDS | -3.30 | 32.60 |
| Sodium monofluorophosphate | MPS | -5.19 | 6.54 |
| 6-aminohexanoic acid | AHA | -8.88 | 11.14 |
| PEG-20 hydrogenated castor oil | POE(20) | -9.07 | 2.52 |
| Potassium nitrate | PNI | -10.26 | 4.37 |
| Sodium fluoride | FLS | -12.83 | 6.44 |
| Sodium N-lauroylsarcosinate | LSS | -28.25 | 4.80 |
| Sodium N-lauroyl-N-methyltaurate | LMT | -30.51 | 3.57 |
| Sodium tetradecene sulfonate | TDS | -44.90 | 3.67 |
| Cocamidopropyl betaine (30% solution) | COB | -52.31 | 12.43 |
