# Supplementary material for: The inhibitory effects of toothpaste and mouthwash ingredients on the interaction between the SARS-CoV-2 spike protein and ACE2, and the protease activity of TMPRSS2 in vitro
Source: PLoS One. 2021 Sep 17;16(9):e0257705. doi: 10.1371/journal.pone.0257705 (PMC8448299; doi:10.1371/journal.pone.0257705)
Supplement: S2 Table — (DOCX) [file pone.0257705.s007.docx]

**S2 Table. Vina score of test ingredients for the refined human TMPRSS2 crystal structure (7MEQ).**

| Ingredients | | Vina Score (kcal/mol) |
| --- | --- | --- |
| Name | Pubchem CID |  |
| Inhibitor | - | −8.1 |
| Gluconic acid | 10690 | −6.0 |
| Tranexamic acid | 5526 | −5.1 |
| N-Lauroylsarcosine | 7348 | −5.1 |
| Dodecyl sulfate | 8778 | −4.8 |
| N-Lauroyl-N-methyltaurine | 61353 | −5.5 |
| (E)-Tetradec-1-ene-1-sulfonic acid | 6437821 | −5.3 |
| 6-aminocaproic acid | 564 | −4.1 |
